# Introgression and isolation contributed to the development of Hungarian Mangalica pigs from a particular European ancient bloodline

**DOI:** 10.1186/1297-9686-45-22

**Published:** 2013-07-01

**Authors:** Ferenc Marincs, János Molnár, Gábor Tóth, Viktor Stéger, Endre Barta

**Affiliations:** 1 Agricultural Biotechnology Center, Szent-Györgyi Albert u. 4, H-2100, Gödöllő, Hungary; 2 Biomi Kft, Szent-Györgyi Albert u. 4, H-2100, Gödöllő, Hungary; 3 Present address: Institute of Enzymology, Hungarian Academy of Sciences, Karolina út 29, H-1113, Budapest, Hungary; 4 Present address: Hungarian Scientific Research Fund, Czuczor u. 10, H-1093, Budapest, Hungary

## Abstract

**Background:**

Mangalica breeds are indigenous to Hungary and their breeding history dates back to about 200–250 years ago. They are fat-type pigs and have a rare curly hair phenotype. The aim of our study was to establish the relationships between these unique breeds and other European breeds.

**Results:**

Based on a core sequence of 382 bp present in 2713 mitochondrial D-loop sequences from pigs belonging to 38 local breeds from nine countries, five cosmopolitan breeds and wild boars from 14 countries, we identified 164 haplotypes. More than half of the 2713 sequences belonged to either four haplotypes characteristic of continental European breeds or two haplotypes characteristic of British/cosmopolitan breeds; each haplotype is present in more than 100 individuals. Most Mangalica individuals belonged either to one of these common continental European haplotypes or to two Mangalica-specific haplotypes that were absent in all other breeds. In addition, we identified the ancestral mitochondrial D-loop signature present in these 2713 sequences and found that ~ 80% carried the European ancient signatures, ANC-Aside and ANC-Cside or their closely related signatures, while most of the remaining sequences carried a modern Asian signature, ANC-Easia. Mangalica individuals carried the ANC-Aside signature, but not the ANC-Cside or ANC-Easia signatures.

**Conclusions:**

In all the Mangalica individuals, a unique ancient European signature was found in the mitochondrial DNA D-loop region, but they belonged almost exclusively to either certain very abundant European or two Mangalica-specific D-loop haplotypes. This indicates that the present-day Mangalica population in Hungary evolved either by introgression of other European breeds and wild boars or via total isolation after the divergence of European ancient porcine bloodlines.

## Findings

There are hundreds of modern pig breeds and many of these are local breeds whereas only a few cosmopolitan breeds are used in the meat industry [1]. The three Mangalica breeds, Blond, Red and Swallow-belly [2] are local, fat-type breeds with characteristic curly hair. They are farmed in Hungary, other successor states of the Austrian-Hungarian Empire and several other countries, and represent a niche pork market. The history of the Mangalica breeds dates back to the late 1700s when both spontaneous and conscious breeding started. Such breeding involved two old Mediterranean pig breeds, Sumadia and Syrmian that originated from the current territories of Serbia and Croatia, and three of the eight old pig breeds that were described in historical Hungary [3,4] i.e. Bakonyi, Szalontai and Alföldi. To date, knowledge on the reproductive biology of the Mangalica breeds is quite extensive [5-9], while their genetics is less studied [10].

Although a number of studies have been conducted to examine the diversity, relationship and introgression between European pigs [11-16], Mangalica breeds have not been examined in this context. Here, we describe a large-scale comparative analysis of mitochondrial DNA (mtDNA) D-loop sequences of Mangalica and other European pig breeds in order to determine the molecular relationships between these breeds and their historical roots.

For this purpose, mtDNA D-loop sequences of 195 Mangalica individuals from a previous study [4] and 2518 additional sequences from the NCBI Genbank [see Additional file 1 and references therein] were combined. The collection covered wild boars from 14 countries, five cosmopolitan breeds, 38 local breeds from nine countries, and eight Mangalica pigs from an unspecified breed [see Additional file 1]. Haplotypes were determined using a 382 bp core sequence shared by all sequences [see Additional file 2]. Within this core sequence, 97 polymorphic positions were identified i.e. 52 transitions, 23 transversions, eight deletions, seven insertions and seven multiple variations, which resulted in 164 haplotypes [see Additional file 3].

Six major haplotypes, each one observed in more than 100 individuals, were identified i.e. HAP07, HAP08, HAP09, HAP13, HAP56 and HAP57. Haplotypes HAP07 and HAP08 correspond to the core European haplotypes C and A, respectively, as described previously [17,18]. In terms of number of individuals, HAP08 was the most abundant haplotype (18.8%), followed by HAP09 (10.4%), HAP07 (9.1%), HAP13 (6.7%), HAP56 (5.6%) and HAP57 (4.3%) and each of these haplotypes was found in 33, 24, 27, 23, 15 and eight breeds and wild boar populations, respectively [see Additional file 4].

Among the 195 Mangalica individuals from our previous study [4], 56 belonged to the two major European haplotypes, HAP08 and HAP13, 91 to the Mangalica-specific haplotypes HAP15 and HAP16, 37 to haplotypes HAP44 and HAP45 and 11 to seven of the minor haplotypes. The eight Mangalica individuals without breed specification collected from another study [see Additional file 1] also belonged to haplotypes HAP08 and HAP13. Taking into consideration that Mangalica pigs separate into three breeds, i.e. Blond, Red and Swallow-belly, it is interesting to note that haplotypes HAP08, HAP15 and HAP45 were present in individuals of all three breeds, while no Red individual carried haplotypes HAP13 and HAP16, and no Swallow-belly individual carried HAP44. Red and Blond Mangalica breeds shared haplotype HAP44 with individuals from the Duroc, Large White and Tamworth breeds, and all three Mangalica breeds shared haplotype HAP45 with Austrian wild boars. Interestingly, the Mangalica breeds had no haplotype in common with the Hungarian wild boars. In this study, individuals associated with haplotypes HAP15 and HAP16 were found only in the Mangalica breeds. Thus, they probably represent Mangalica-specific maternal lineages.

**Figure 1:**
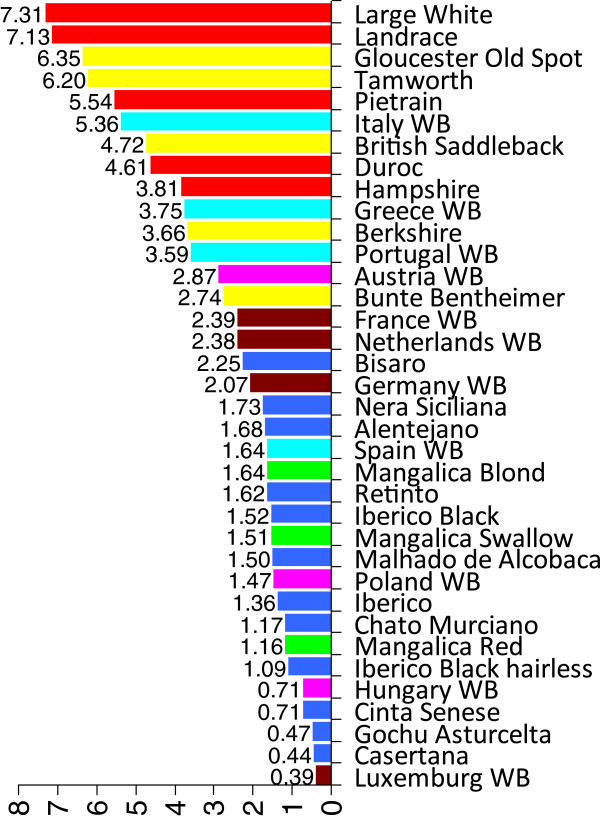
**Within-breed/population diversity of domestic pigs and wild boars.** Values over the bars indicate “average number of pair-wise differences within populations” values; breeds and wild boar populations of different geographical origin are shown by different colours: brown = Western-Europe wild boars, yellow = Western-Europe local pigs, light blue = Southern-Europe wild boars, dark blue = Southern-Europe local pigs, magenta = Central-Europe wild boars, red = cosmopolitan breeds and green = Mangalica breeds.

We computed the F_ST_ and Nei’s genetic distance indices for breeds for which mtDNA D-loop sequences were available for ten or more individuals. The results revealed that the within-breed diversity of the Mangalica breeds was similar to that of local Spanish and Italian breeds and that the Mangalica breeds were more homogeneous than the cosmopolitan and British local breeds and certain South-European wild boars (Figure 1). The Swallow-belly and Blond Mangalica breeds were genetically closer to each other, than to the Red Mangalica breed as shown by the pair-wise F_ST_ and Nei’s distance values (Table 1). The Red Mangalica breed was genetically closer to continental European breeds and wild boars than the Blond and Swallow-belly Mangalica breeds (Table 1). With the exception of the comparison between the German wild boar population and the Red Mangalica breed, all F_ST_ and Nei’s pair-wise distance values were significant, at the *P* = 0.05 level, between any one of the three Mangalica breeds and any other breed involved in this study (data not shown).

**Table 1:** Genetic distance values between pig breeds

**Breed **^**1**^	**F**_**ST**_	**Nei's distance**
BM vs. RM	0.39	0.97
BM vs. SM	0.09	0.16
RM vs. SM	0.29	0.56
	**Mean F**_**ST**_^**2**^	**Mean Nei's distance**^**2**^
BM vs. non-M	0.52 ± 0.13	2.82 ± 2.21
SM vs. non-M	0.46 ± 0.15	2.34 ± 2.22
RM vs. non-M	0.33 ± 0.20	1.70 ± 2.45
non-M vs. non-M	0.33 ± 0.21	2.16 ± 2.48

^1^*BM* Blond Mangalica, *SM* Swallow-belly Mangalica, *RM* Red Mangalica, non-M = other breeds; ^2^mean values were calculated by averaging the pair-wise values between the breeds.

An analysis on 208 European archaeological pig specimens identified five mtDNA D-loop signatures: ANC-Aside, ANC-Cside, ANC-Italy, ANC-Y1-6A and ANC-Y2-5A [19]. Of these 208 samples, 109 (52.4%) carried ANC-Aside and 65 (31.25%) ANC-Cside, while the remaining 34 samples (16.35%) had one of the other three signatures. In a previous study, we showed that 197 (97%) of the 203 Mangalica individuals analysed (all included in the haplotype analysis described here) also carry the ANC-Aside signature, while the remaining six individuals carry signatures that are phylogenetically very close to ANC-Aside [4]. To compare other European breeds with the Mangalica breeds and to determine the relationships to their ancestors, we identified the ancient signature of the remaining 2518 individuals of our current study and found that the ANC-Aside and ANC-Cside signatures were present in modern pigs at frequencies (42.5% and 33.5%, respectively) similar to those in the ancient pig specimens. In contrast, the frequency of the ANC-Italy, ANC-Y1-6A, and ANC-Y2-5A signatures dropped to less than 1.0% each. We note here that the ANC-Easia signature, which was not found in the European archaeological specimens [19], was observed in 455 of the 2713 modern pigs that we analysed. This is due, in part, to the introgression of Asian pig breeds into cosmopolitan and local British breeds, since 92.1% of the individuals with the ANC-Easia signature belong to these breeds.

Our analysis revealed that individuals that carried the major European haplotypes HAP07 or HAP09, HAP08 or HAP13 and HAP56 or HAP57 also carried ANC-Cside, ANC-Aside or ANC-Easia signatures, respectively [see Additional file 4]. It is important to note, that both the ANC-Aside and ANC-Cside signatures were present in several breeds and wild boar populations and consequently they can be characterised by more than one haplotype.

While Mangalica breeds can belong to several haplotypes, almost 50% of the breeds analyzed displayed only two haplotypes, HAP15 and HAP16, which were not present in any of the other breeds and wild boars studied. Furthermore, all 203 Mangalica individuals in this study carried the ANC-Aside signature.

Both the ANC-Aside and ANC-Cside signatures and the haplotypes HAP08 and HAP07 that carry them differ by one nucleotide substitution. Due to the difference between the lengths of the signature and the haplotype sequences [see Additional file 2], the calculated divergence time between ANC-Aside and ANC-Cside signatures, and between HAP08 and HAP07 is ~98 000 and ~190 000 YBP, respectively. This in good agreement with previously reported results [17,20] and clearly indicates that the time of divergence between the European porcine maternal lineages carrying the ANC-Aside and ANC-Cside signatures is much older than the age of the oldest European archaeological specimen [19] and the predicted time of wild boar domestication [21].

**Figure 2:**
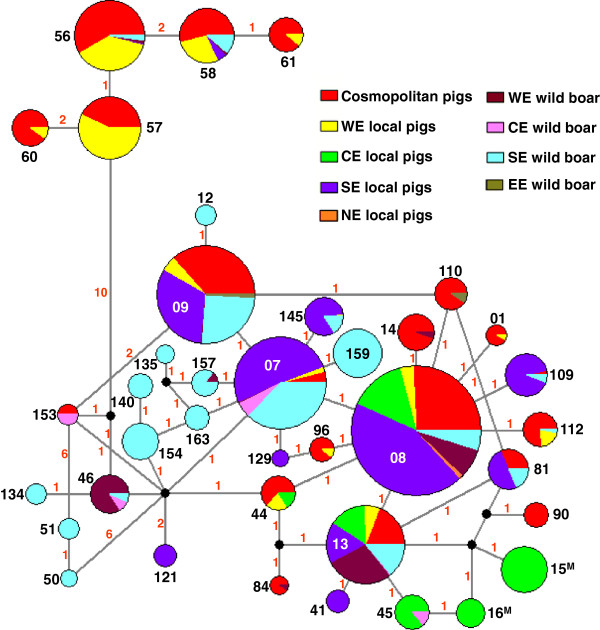
**Distribution of pig breeds in the median-joint network of haplotypes.** The size of the nodes is proportional to the number of individuals with each haplotype; black or white numbers indicate the haplotype numbers and red numbers indicate the number of mutations between the nodes; for clarity, only the haplotypes for which 10 or more individuals carry the haplotype are shown, and the length of the connectors is not proportional to the number of mutations between the nodes; WE = Western Europe (Belgium, France, Germany, Luxemburg, The Netherlands, United Kingdom), CE = Central Europe (Austria, Croatia, Hungary, Poland), SE = Southern Europe (Greece, Italy, Portugal, Spain), NE = Northern Europe (Finland, Sweden), EE = Eastern Europe (Bulgaria); superscript M denotes Mangalica-specific haplotypes.

There are several possible explanations for the unique signature/haplotype characteristics of the Mangalica breeds. One hypothesis is that ancestors of the current Mangalica population in Hungary that carried the ANC-Aside signature and of the European wild boar populations that carried the ANC-Cside signature became geographically isolated during prehistoric times. Another possibility is that the ANC-Aside signature evolved from the ANC-Cside signature in the Mangalica ancestor species after isolation. It cannot be excluded that either the population bottleneck that occurred in the Mangalica population during the 1960’s [22] or breeding practices selected against the ANC-Cside signature if present in the Mangalica population. However, given the presence of Mangalica-specific haplotypes, the isolation theory is the most likely. The network of haplotypes (Figure 2) suggests that the breed-specific haplotypes HAP16 and HAP15 evolved from HAP45 by one nucleotide substitution and from either HAP13 or HAP16 by two nucleotide substitutions, respectively. Consequently, the time of divergence between these haplotypes and of their possible isolation dates back to ~190 000 and ~380 000 YBP. The disappearance of Western- and Southern-European and Austrian wild boars and parallel evolution of HAP45 to HAP16 and HAP13 to HAP15, suggest that isolation might have occurred by a west-to-east migration of Mangalica ancestor species. This is also supported by the fact that certain Mangalica individuals and Austrian wild boars share haplotype HAP45.

In summary, we conclude that the present-day Mangalica population in Hungary may have originated by two different mechanisms: either introgression of common European bloodlines into the Mangalica breeds or by total isolation of some Mangalica ancestor species, as suggested by the presence of both common European and Mangalica-specific mtDNA D-loop haplotypes in the population.

## Competing interests

The authors declare that they have no competing interests.

## Authors’ contributions

FM conceived the study, performed the haplotype and network analyses, and drafted the manuscript; JM performed the diversity analyses; GT participated in data analyses and helped to draft the manuscript; VS performed data collection and management; EB helped to analyse data and to draft the manuscript. All authors read and approved the final manuscript.

## Supplementary Material

Additional file 1 **Breeds used in this study with their location and number of individuals.** The file provides information about the breeds, their location and GenBank accession number of the individuals’ mitochondrial D-loop sequence with references [4,14,17,18,20,23-33]. The haplotypes and ancient signatures of the individuals as determined in this study are also provided. Click here for file

Additional file 2 **Materials and methods.** The file provides information about the methods with references [23,34-41] that were used for analysis of the D-loop sequences of the individuals in Additional file 1. Click here for file

Additional file 3 **Polymorphic positions in the mtDNA D-loop haplotypes identified in this study.** The table shows the polymorphic positions in the determined haplotypes relative to the reference sequence AJ002189 [34]. Vertical numbers in row 2 indicate the nucleotide position in the reference sequence AJ002189; empty cell in row 2 indicates the presence of an insertion in the haplotype as compared to the reference sequence AJ002189; empty cells in the table indicate nucleotides that are identical with the reference and cells with lines indicate deletions. Click here for file

Additional file 4 **Distribution of haplotypes and ancient signatures among European modern pig breeds and wild boars.** The file provides information about the abundance of the D-loop haplotypes in breeds and pig types and of the ancient signatures in breeds, and about the correlation between the major European haplotypes and their ancient signature. Click here for file
