# Supplementary material for: Introgression and isolation contributed to the development of Hungarian Mangalica pigs from a particular European ancient bloodline
Source: Genet Sel Evol. 2013 Jul 1;45(1):22. doi: 10.1186/1297-9686-45-22 (PMC3704957; doi:10.1186/1297-9686-45-22)
Supplement: Additional file 2 — Materials and methods. The file provides information about the methods with references [23,34-41] that were used for analysis of the D-loop sequences of the individuals in Additional file 1. [file 1297-9686-45-22-S2.doc]

**Materials and methods**

To determine the haplotypes, sequences were aligned and then trimmed to a 382 bp long common sequence (Sequence 1), corresponding to the region between positions 15 483 and 15 858 in the pig mtDNA reference sequence AJ002189 [34]. Haplotypes were determined from the aligned sequences by manual sorting of the same sequences. To identify the ancient signature of the 2713 sequences, they were trimmed to a core signature sequence (Sequence 2), corresponding to the D‑loop between positions 15 520 and 15 593 in the reference pig mtDNA sequence AJ002189 [34] and then aligned with the five ancient mtDNA D‑loop haplotypes (signatures) identified in European archaeological pig specimens [19]. For sequence alignments, we used the software Geneious version 5.3.6 with default “Geneious Alignment” settings (Cost matrix: 65% similarity, Gap open penalty: 12, Gap extension penalty: 3, Alignment type: Global alignment with free end gaps) [35], then the aligned sequences were trimmed. Pair-wise genetic differences within breeds and pair-wise indices between breeds, such as fixation index (F_ST_), Nei’s and Reynold’s distances [36-38] were calculated and visualised using the software Arlequin version 3.5 [39] by setting the number of permutations to 1000 and the significance level to 0.05 for *P*‑value calculations. The between‑breed mean values for the fixation index and Nei’s distance were calculated in Excel. Median joining networks were constructed using the software Network v4.6 [40]. Divergence time was calculated using the equation *T* = *K*/2*r* [23], where *K* is the substitution/site value (number of substitutions divided by length of the sequence) and *r* is substitution rate (1.37 × 10^-8^ substitution/site/year) for the mammalian mitochondrial genome [41]. The previously analysed sequences [4] were submitted to the NCBI nucleotide database under accession numbers JX546298-JX546572 and PopSet 407196600.

Sequence 1 and 2 are between positions 15 483 and 15 858 and between 15 520 and 15 593, respectively, in the pig mtDNA reference sequence AJ002189 [34].

Sequence 1

5’TTAATTACTATCTTTAAAACAAAAAAACCCATAAAAATTGCGCACAAACATACAAATATGTGACCCCAAAAATTTAACCATTGAAAACCAAAAAATCTAATATACTATAACCCTATGTACGTCGTGCATTAATTGCTAGTCCCCATGCATATAAGCATGTACATATTATTATTAATATTACATAGTACATATTATTATTGATCGTACATAGCACATATCATGTCCAAATAATTCCAGTCAACATGCATATCACCACCACTAGATCACGAGCTTAATTACCATGCCGCGTGAAACCAGCAACCCGCTTGGCAGGGATCCCTCTTCTCGCTCCGGGCCCATAAACCGTGGGGGTTTCTATTGATGAACTTTAACAGGC 3’

Sequence 2

5’ TTGCGCACAAACATACAAATATGTGACCCCAAAAATTTAACCATTGAAAACCAAAAAAT

CTAATATACTATAAC 3’
